# Supplementary material for: A new preprocedural predictive risk model for post-endoscopic retrograde cholangiopancreatography pancreatitis: The SuPER model
Source: eLife. 2025 Jan 17;13:RP101604. doi: 10.7554/eLife.101604 (PMC11741517; doi:10.7554/eLife.101604)
Supplement: Supplementary file 1. — PEP, post-endoscopic retrograde cholangiopancreatography pancreatitis. a Patients with missing data for variables selected in the risk score model were removed. [file elife-101604-supp1.docx]

**Supplementary file 1.**Risk classification and unpredictable intraprocedural risk factors for PEP (multivariate logistic regression).

| Cohort | Factor | OR | 95% CI | *P* value |
| --- | --- | --- | --- | --- |
| All | Risk classification | 4.17 | 2.76-6.30 | < 0.01 |
| (n = 2017) ^a^ | Precut sphincterotomy (n = 52) | 1.26 | 0.54-2.96 | 0.59 |
|  | Inadvertent pancreatic duct cannulation (n = 349) | 0.83 | 0.49-1.42 | 0.50 |
| Development | Risk classification | 5.97 | 3.21-11.1 | < 0.01 |
| (n = 1012) ^a^ | Precut sphincterotomy (n = 26) | 0.33 | 0.04-2.57 | 0.29 |
|  | Inadvertent pancreatic duct cannulation (n = 193) | 0.63 | 0.30-1.35 | 0.23 |
| Validation | Risk classification | 2.97 | 1.71-5.16 | < 0.01 |
| (n = 1005) ^a^ | Precut sphincterotomy (n = 26) | 2.57 | 0.93-7.08 | 0.07 |
|  | Inadvertent pancreatic duct cannulation (n = 156) | 1.05 | 0.49-2.27 | 0.90 |

PEP, post-endoscopic retrograde cholangiopancreatography pancreatitis; OR, odds ratio; CI, confidence interval.

^a^ Patients with missing data for variables selected in the risk score model were removed.
