# Supplementary material for: A new preprocedural predictive risk model for post-endoscopic retrograde cholangiopancreatography pancreatitis: The SuPER model
Source: eLife. 2025 Jan 17;13:RP101604. doi: 10.7554/eLife.101604 (PMC11741517; doi:10.7554/eLife.101604)
Supplement: Supplementary file 2. — PEP, post-endoscopic retrograde cholangiopancreatography pancreatitis. [file elife-101604-supp2.docx]

**Supplementary file 2.**　Risk of PEP following implantation of pancreatic stents (logistic regression).

| Cohort | Pancreatic stent | N | OR | 95% CI | *P* value |
| --- | --- | --- | --- | --- | --- |
| All | All | 310/2074 | 3.32 | 2.27-4.85 | < 0.01 |
|  | Prophylactic | 191/2074 | 3.35 | 2.17-5.16 | < 0.01 |
| Development | All | 169/1037 | 2.75 | 1.62-4.67 | < 0.01 |
|  | Prophylactic | 107/1037 | 2.88 | 1.58-5.25 | < 0.01 |
| Validation | All | 141/1037 | 4.06 | 2.36-7.01 | < 0.01 |
|  | Prophylactic | 84/1037 | 4.01 | 2.14-7.52 | < 0.01 |

PEP, post-endoscopic retrograde cholangiopancreatography pancreatitis; OR, odds ratio; CI, confidence interval.
